# The Resection Ability of Low‐power Pure‐cut Hot Snare Polypectomy for Nonpedunculated Colorectal Polyps Smaller Than 10 mm: A Single‐center Prospective Study

**DOI:** 10.1002/deo2.70450

**Published:** 2026-10-12

**Authors:** Ryo Mizuno, Hidenori Kimura, Ryoji Kushima, Akifumi Notsu, Atsushi Nishida, Osamu Inatomi, Takuji Iwashita

**Affiliations:** ^1^ Department of Medicine Division of Gastroenterology Shiga University of Medical Science Shiga Japan; ^2^ Department of Medicine Division of Digestive Endoscopy Shiga University of Medical Science Shiga Japan; ^3^ Department of Pathology Shiga University of Medical Science Shiga Japan; ^4^ Clinical Research Center Shizuoka Cancer Center Shizuoka Japan

**Keywords:** endoscopic mucosal resection, hot snare polypectomy, low‐power pure‐cut current, nonpedunculated colorectal polyps, small colorectal polyps

## Abstract

**Objectives:**

Small colorectal polyps sometimes include high‐grade adenomas or carcinomas that require endoscopic resection with electrocautery. Recent studies have reported the usefulness of low‐power pure‐cut hot snare polypectomy (LPPC‐HSP) as a less invasive technique. However, the actual resection depth of LPPC‐HSP has not been fully investigated. Therefore, this study aimed to evaluate the resection ability of LPPC‐HSP for small colorectal polyps.

**Methods:**

In this single‐arm prospective observational study, patients scheduled to undergo LPPC‐HSP for nonpedunculated colorectal polyps smaller than 10 mm were enrolled. The primary outcome was the rate of sufficient vertical R0 resection (SVR0), defined as an R0 resection with a sufficient submucosal layer beneath the whole lesion.

**Results:**

A total of 95 patients with 150 lesions were included. The en bloc and R0 resection rates were 100% and 83%, respectively. The SVR0 rate was 76.7% (90% confidence interval: 70.3%–82.2%). All specimens contained the muscularis mucosa, and 132 lesions (88%) contained submucosal tissue. The median maximum submucosal thickness was 1331 µm (interquartile range, 736–2044 µm). No bleeding, perforation, or other serious adverse events were observed.

**Conclusions:**

LPPC‐HSP demonstrated acceptable vertical resection capability. LPPC‐HSP may be applicable for colorectal polyps smaller than 10 mm with potential malignancy, providing resection depth comparable to that of conventional electrocautery‐based methods.

**Trial Registration**: University Hospital Medical Network Clinical Trials Registry: UMIN000054732

## 1 Introduction

Detection and resection of adenomatous colorectal polyps by colonoscopy have been shown to reduce the incidence and mortality of colorectal cancer [1, 2]. Colorectal polyps smaller than 10 mm are often encountered during routine colonoscopy in daily clinical practice [3]. For small nonpedunculated polyps, cold snare polypectomy (CSP) is widely performed as the standard treatment [4, 5, 6, 7, 8]. However, some colorectal polyps smaller than 10 mm harbor high‐grade dysplasia or intramucosal carcinoma [5, 9, 10, 11]. When high‐grade adenomas or carcinomas are suspected, CSP cannot entirely excise the muscularis mucosa [12, 13], and the use of electrocautery during endoscopic resection is recommended. Endoscopic electrocautery resection methods, including hot snare polypectomy (HSP) and endoscopic mucosal resection (EMR), are widely used; however, they are associated with a non‐negligible risk of adverse events, including bleeding and perforation due to deep thermal injury. The reported intraoperative perforation rates are 0.02% for HSP and 0.09% for EMR, while delayed bleeding rates are 1.3% and 1.4%, respectively [14].

Recently, low‐power pure‐cut HSP (LPPC‐HSP) has been developed [15, 16, 17]. This technique is expected to reduce thermal damage to the muscle layer by using a pure‐cut current without coagulation and reducing power [16]. A retrospective, propensity score–matched study showed that LPPC‐HSP had comparable resection outcomes, including en bloc resection and R0 resection, to conventional EMR, and the incidence of adverse events with LPPC‐HSP was lower than with conventional EMR [17], suggesting that LPPC‐HSP may be a safe and effective treatment option for small polyps. However, to determine whether this technique is appropriate for lesions such as high‐grade dysplasia and intramucosal carcinoma that can extend into the muscularis mucosa, it is necessary to examine the actual resection depth of LPPC‐HSP; however, no such studies have been conducted for polyps smaller than 10 mm. Thus, we investigated the treatment outcomes of LPPC‐HSP, including resection depth, for nonpedunculated colorectal polyps smaller than 10 mm.

## 2 Methods

### 2.1 Study Design and Patients

This single‐center, single‐arm, prospective observational study was initiated at our hospital in July 2024. The inclusion criteria were patients aged ≥18 years who were scheduled to undergo LPPC‐HSP for at least one nonpedunculated colorectal polyp smaller than 10 mm. LPPC‐HSP was performed for nonpedunculated colorectal polyps smaller than 10 mm if at least one of the following advanced histological endoscopic findings was observed: surface redness, white spots, nonpolypoid growth, loss of lobulation, heterogeneity, irregular capillary pattern on narrow‐band imaging [10], or a low‐grade adenoma could not be diagnosed with high confidence. The exclusion criteria were inflammatory bowel disease, polyposis syndrome, blood coagulation dysfunction, active infectious disease, dialysis, pregnancy, and inability to discontinue antithrombotic agents. This study was approved by the Institutional Review Board of Shiga University of Medical Science (No. R2024‐013) on May 27, 2024. This study was registered with the University Hospital Medical Network Clinical Trials Registry (UMIN000054732). Written informed consent was obtained from all the participants.

### 2.2 Endoscopic Procedures and Patient Management

All procedures were performed using high‐definition colonoscopes (PCF‐H290ZI or CF‐EZ1500D; Olympus Corporation, Tokyo, Japan, or EC760‐ZP‐V; Fujifilm Corporation, Tokyo, Japan) with disposable rubber distal attachments (MAJ‐1990, 1991 or D‐201‐12704, 14304; Olympus Corporation, Tokyo, Japan). A standard electrosurgical generator (VIO300D or VIO3; ERBE) and a 10‐ or 15‐mm oval snare with a wire diameter of 0.30 mm (SnareMaster Plus S; Olympus Corporation, Tokyo, Japan) were used.

All procedures were performed by nine gastrointestinal fellows and 14 experts (certified by the Japan Gastroenterological Endoscopy Society). Experienced endoscopists supervised the gastrointestinal fellows when the polypectomy was performed. The Paris classification was used to define polyp morphology [18]. Lesion size was measured before resection using the tip of the snare sheath, deployed snare, non‐traumatic tube, or ruler. For LPPC‐HSP, a 10‐ or 15‐mm oval snare was used without submucosal injection, and the polyps were resected using a low‐power pure‐cut current (Autocut; VIO300D: effect, 1 [10 W]; VIO3: effect, 0.4) [16, 17] (Figure 1). We routinely performed air deflation before snaring to capture the lesion together with sufficient surrounding normal mucosa. Prophylactic clipping was performed based on the endoscopist's preference. Resected specimens were retrieved using button‐attached suction forceps or nets. After fixation in 10% formalin, specimens were sent to the pathology department for histological assessment. The fixed specimens were sectioned consecutively into 2–3 mm slices to obtain adequately sectioned histological specimens. They were then embedded in paraffin, sectioned into 3–4 µm slices, and stained with hematoxylin and eosin for pathological evaluation. Histological diagnoses were made according to the Japanese Classification of Colorectal Carcinoma [19], and assessments were conducted by experienced pathologists. At our institution, patients treated with LPPC‐HSP were admitted for two days to monitor for possible adverse events. Follow‐up was conducted on an outpatient basis 2–4 weeks after the colonoscopy.

**FIGURE 1:**
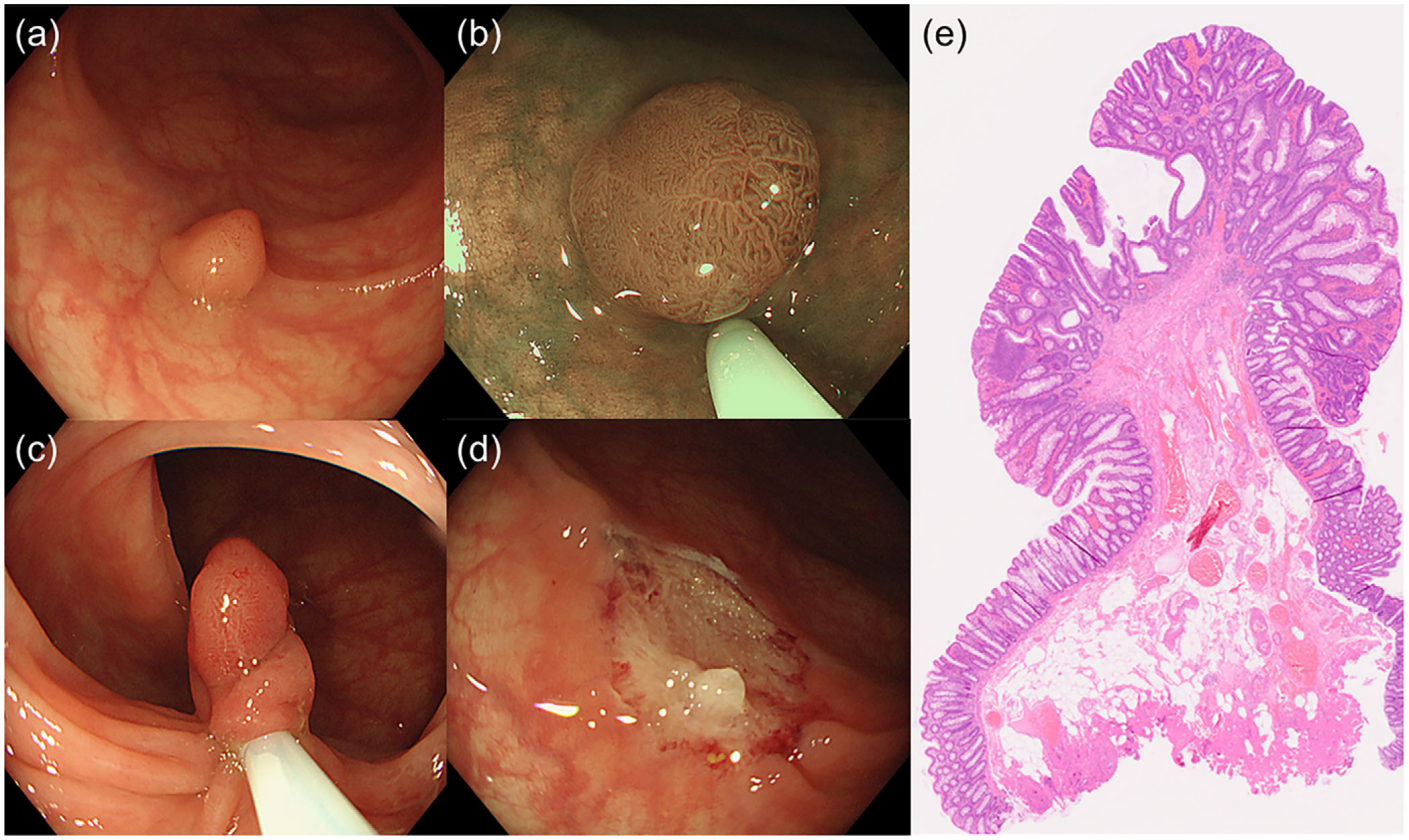
LPPC‐HSP procedure. (a) An 8‐mm sessile polyp located in the sigmoid colon. (b) The NBI surface pattern was heterogeneous, suggesting advanced histology. (c) The polyp was resected by LPPC‐HSP. (d) The ulcer after LPPC‐HSP. (e) Low‐grade adenoma with tumor‐free margins. LPPC‐HSP, low‐power pure‐cut hot snare polypectomy; NBI, narrow‐band imaging.

### 2.3 Outcomes and Definition

The primary outcome was the rate of sufficient vertical R0 resection (SVR0) [20], defined as an R0 resection that included a sufficient submucosal layer beneath the whole lesion on adequately sectioned histological specimens. Lesions with an unevaluable horizontal resection margin, including specimen damage or inadequate sectioning, were classified as non‐R0. Even in colorectal polyps smaller than 10 mm, high‐grade adenomas or intramucosal cancers have the potential to eventually progress and invade the muscularis mucosa. Complete resection of the muscularis mucosa is essential for treating such lesions. Since specimens, including submucosal tissue, enabled reliable pathological diagnosis, including invasion depth, even in carcinomas with submucosal invasion, we regarded SVR0 as significant and set it as the primary outcome. Procedure time was defined as the duration from the insertion of the snare into the channel to the completion of LPPC resection. En bloc resection was defined as the complete removal of the lesion in one piece during endoscopic resection. R0 resection was defined as an en bloc resection with pathologically negative margins. Immediate bleeding was defined as bleeding that did not diminish within 30 s after endoscopic resection and required hemostatic interventions, including endoscopic hemostats, interventional radiology, or surgery [15, 16, 17, 21]. Delayed bleeding was defined as bleeding requiring hemostatic intervention or blood transfusion due to the appearance of hematochezia within 14 days after resection. Intraprocedural perforation was defined as the direct endoscopic appearance of peritoneal fat during the procedure. Delayed perforation was defined as abdominal pain in conjunction with computed tomography findings of free air. Two gastroenterologists and ten experienced pathologists discussed and evaluated SVR0, the presence of the entire muscularis mucosa and submucosal tissue beneath the lesion, maximum submucosal thickness, submucosal thickness at the center, and the margin of the specimens (Figures 2 and 3). Specimen damage cases were excluded from submucosal thickness measurement. The submucosal thickness was measured perpendicular to the muscularis mucosa. When only the muscularis mucosa was included in the specimen, the submucosal thickness was defined as 0 µm. All cases were analyzed using NIS Elements D (Nikon Solutions Co., Tokyo, Japan), based on the largest histological section of each lesion.

**FIGURE 2:**
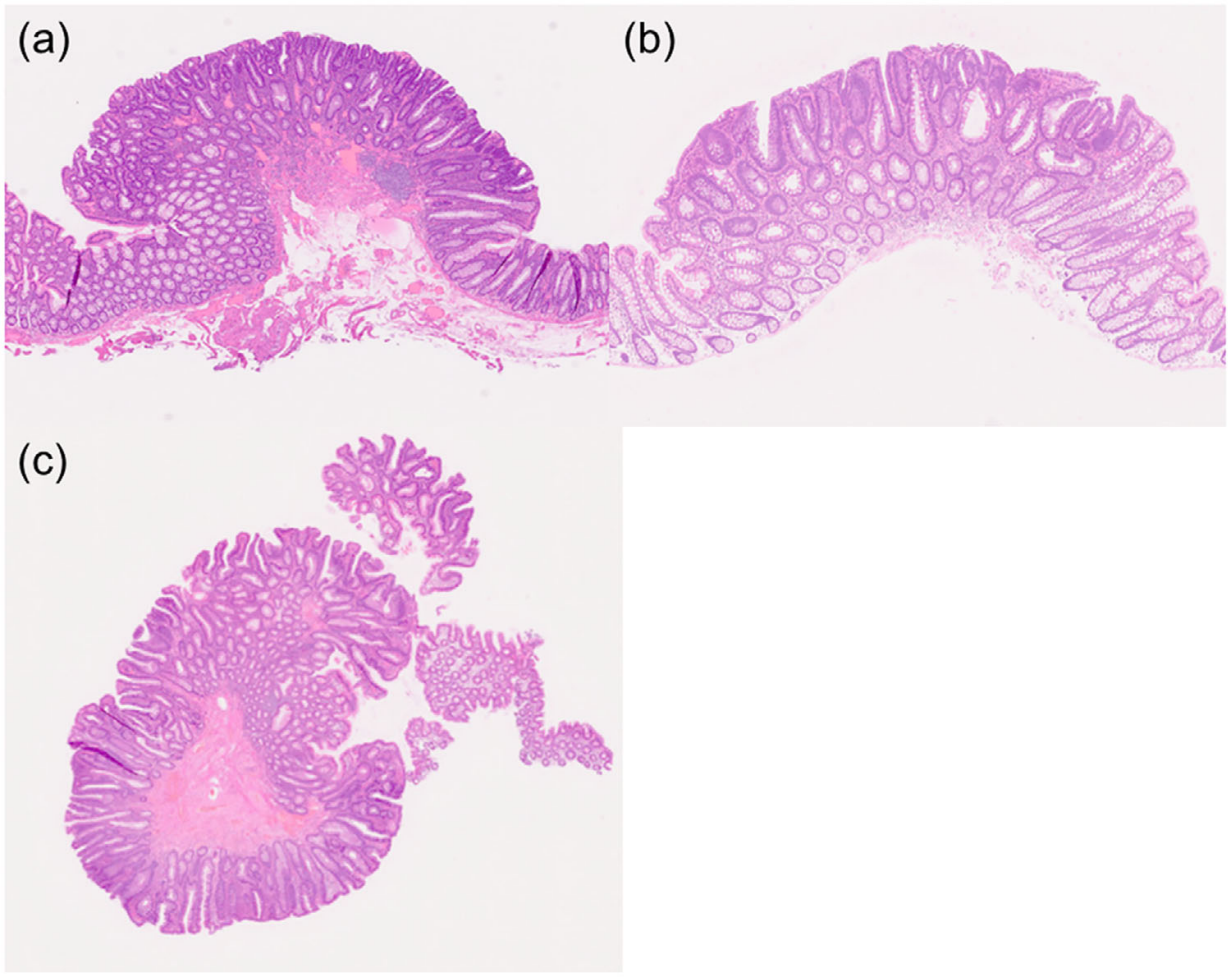
Pathological evaluation of sufficient vertical R0 resection. (a) Sufficient vertical R0 resection (SVR0). (b) R0 resection without submucosal tissue. (c) Non‐R0 resection.

**FIGURE 3:**
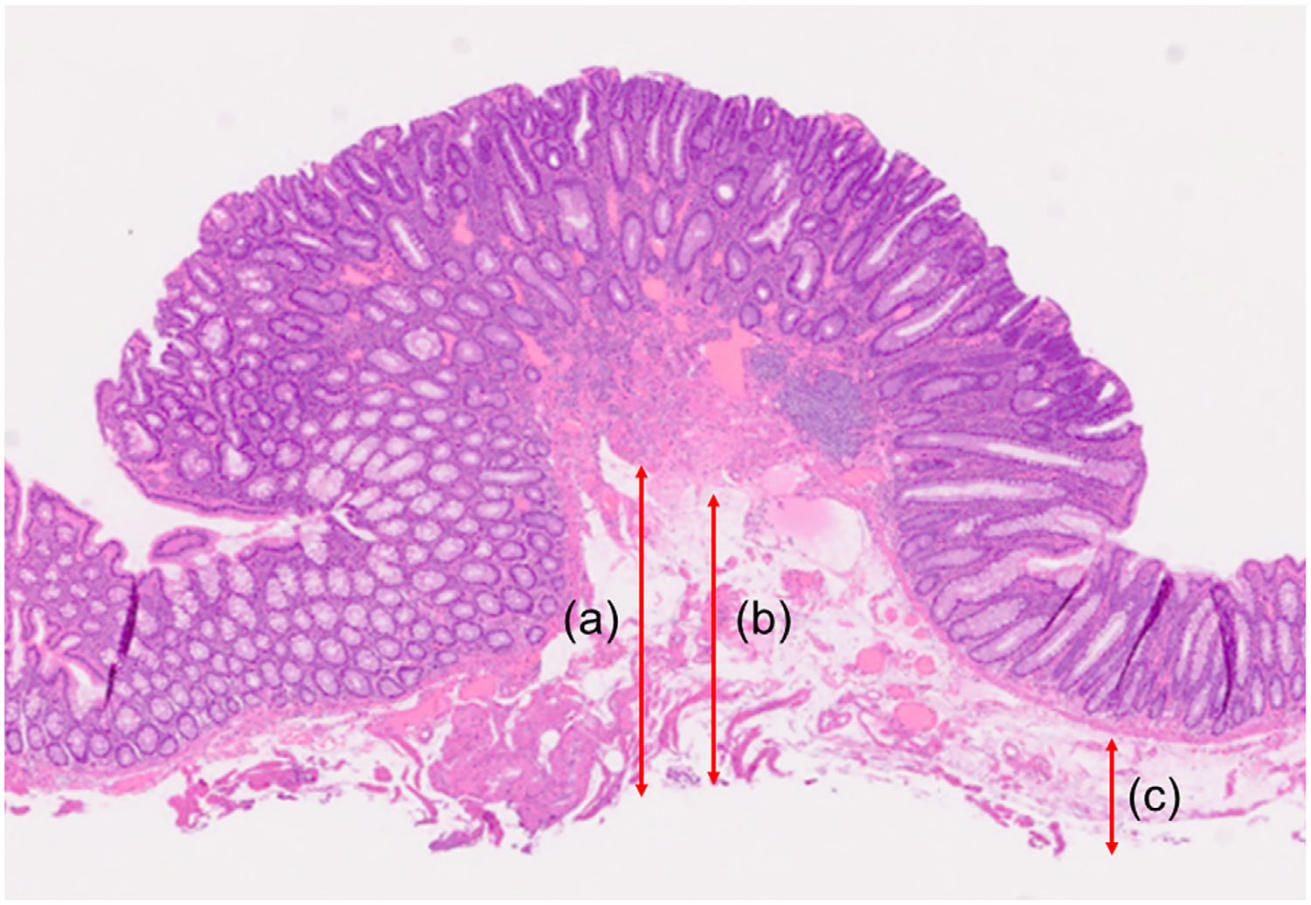
Measurement of submucosal thickness. (a) Maximum submucosal thickness. (b) Submucosal thickness at the center of the specimen. (c) Submucosal thickness at the margin of the specimen.

### 2.4 Statistical Analysis

Based on previous studies [22, 23, 24] that reported R0 resection rates of EMR ranging from 62% to 87.3%, and the rate of submucosal tissue containment for small colorectal polyps of approximately 92%, the expected rate of SVR0 was set at 70%. With a non‐inferiority margin of 10%, the predefined threshold rate was set at 60%. Assuming a one‐sided α error of 0.05 and β error of 0.2, the required sample size was calculated to be 143 cases. Considering potential dropouts, we enrolled 150 cases. The primary endpoint was evaluated against a predefined threshold of 60% using a one‐sided exact binomial test. Subgroup analyses were performed according to snare size and prophylactic clip to compare treatment outcomes. Medians with interquartile ranges (IQRs) were used for continuous variables, and numbers with percentages were used for categorical variables. All statistical analyses were performed using the EZR software version 1.68 (Saitama Medical Center, Jichi Medical University, Saitama, Japan).

## 3 Results

### 3.1 Patients and Lesions

A patient flow diagram is shown in Figure 4. Finally, 95 patients (150 lesions) were analyzed in this study. Baseline characteristics are summarized in Table 1. The median patient age was 74 years (IQR: 68–78). Seventy‐four patients (78%) were men, and 21 (22%) were taking antithrombotic agents. The median lesion size of the polyps was 7 mm (IQR: 6–8).

**FIGURE 4:**
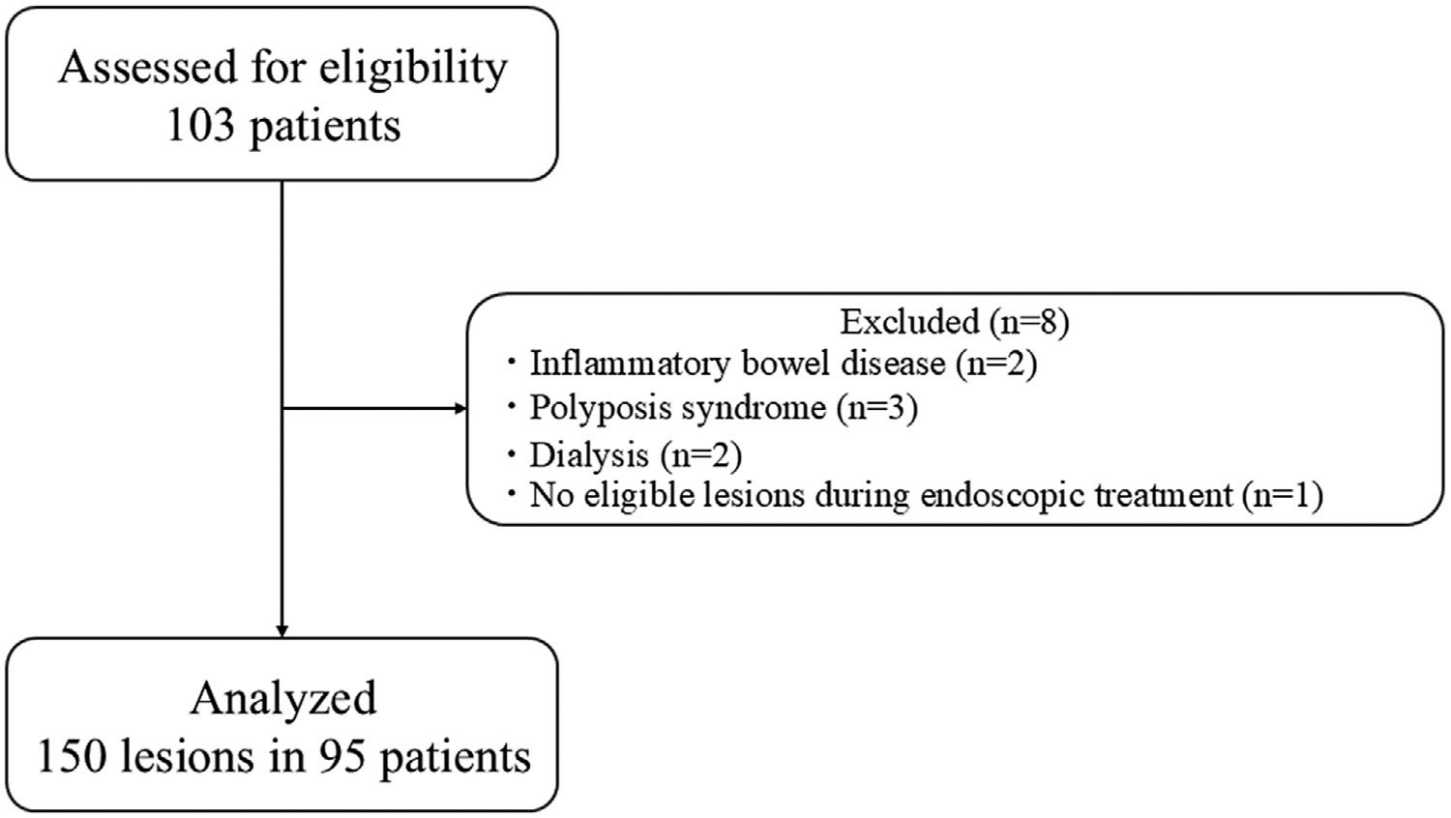
Patient flow diagram.

**TABLE 1:** Baseline characteristics.

	LPPC‐HSP
**Patients**	***n* = 95**
Age, years (IQR)	74 (68‐78)
Male sex, *n* (%)	74 (78)
Antithrombotic agents, *n* (%)	21 (22)
Two antithrombotic agents	2 (2.1)
One antithrombotic agent	19 (20)
**Lesions**	***n* = 150**
Size, mm (IQR)	7 (6–8)
Location, *n* (%)	
Cecum	10 (6.7)
Ascending colon	32 (21)
Transverse colon	38 (25)
Descending colon	17 (11)
Sigmoid colon	46 (31)
Rectum	7 (4.7)
Macroscopic type, *n* (%)	
0‐Is	130 (87)
v0‐IIa	20 (13)

Abbreviations: IQR, interquartile range; LPPC‐HSP, hot snare polypectomy with low‐power pure‐cut current.

### 3.2 Treatment Outcomes

Treatment outcomes are summarized in Table 2. All lesions were successfully resected using LPPC‐HSP. The median procedure time was 65 s (IQR: 50–91). Gastrointestinal fellows performed LPPC‐HSP for 48 lesions (32%). En bloc resection was achieved in all cases. Prophylactic clipping was performed for 38 lesions (25%). No bleeding, perforation, or other adverse events were observed.

**TABLE 2:** Treatment outcomes.

	LPPC‐HSP
Lesions	*n* = 150
Procedure time, seconds (IQR)	65 (50–91)
Gastrointestinal fellows performed, *n* (%)	48 (32)
Completion of LPPC‐HSP, *n* (%)	150 (100)
En bloc resection, *n* (%)	150 (100)
Prophylactic clipping, *n* (%)	38 (25)
Immediate bleeding, *n* (%)	0 (0)
Delayed bleeding, *n* (%)	0 (0)
Intraprocedural perforation, *n* (%)	0 (0)
Delayed perforation, *n* (%)	0 (0)
Abdominal symptoms, *n* (%)	0 (0)
Unscheduled physician visit, *n* (%)	0 (0)

Abbreviations: IQR, interquartile range; LPPC‐HSP, hot snare polypectomy with low‐power pure‐cut current.

### 3.3 Pathological Evaluation

Histopathological findings are summarized in Table 3. No specimens were fixed by pinning. R0 resection was achieved in 124 lesions (83%). All specimens contained the muscularis mucosa, and 132 lesions (88%) contained submucosal tissue. The primary outcome, the SVR0 rate, was 76.7% (115/150; 90% confidence interval [90% CI]: 70.3%–82.2%). The lower limit of the 90% CI exceeded the predefined threshold of 60%, and the primary endpoint was met (one‐sided *p* < 0.001). Of the 150 lesions, four specimen‐damaged cases were excluded from submucosal thickness measurement. The median maximum submucosal thickness was 1331 µm (IQR: 736–2044) overall, 1224 µm (IQR: 607–2032) at the center, and 849 µm (IQR: 509–1282) at the margin.

**TABLE 3:** Pathological evaluation.

	LPPC‐HSP
Lesions	*n* = 150
Pin fixation	0 (0)
R0 resection, *n* (%)	124 (83)
Non‐R0 resection, *n* (%)	26 (17)
Specimen damage	4 (2.7)
Inadequate sectioning	14 (9.3)
pHMX/1	8 (5.3)
pVMX/1	0 (0)
Sufficient vertical R0 resection (SVR0), *n* (%)	115 (77)
Pathology, *n* (%)	
Low‐grade adenoma	133 (89)
High‐grade adenoma	11 (7.3)
Villous adenoma	1 (0.7)
Sessile serrated lesion	1 (0.7)
Traditional serrated adenoma	3 (2.0)
Hyperplastic polyp	1 (0.7)
Presence of muscularis mucosa within the resected specimen, *n* (%)	150 (100)
Presence of submucosal tissue within the resected specimen, *n* (%)	132 (88)
Maximum thickness of submucosal tissue within the resected specimen, µm (IQR)	1331 (736–2044)
Thickness of submucosal tissue within the resected specimen at the center, µm (IQR)	1224 (607–2032)
Thickness of submucosal tissue within the resected specimen at the margin, µm (IQR)	849 (509–1282)

Abbreviations: IQR, interquartile range; LPPC‐HSP, hot snare polypectomy with low‐power pure‐cut current. Specimen damage cases were excluded from submucosal thickness measurement.

### 3.4 Subgroup Analysis

Subgroup analyses according to prophylactic clip and snare size were performed for baseline characteristics, treatment outcomes, and pathological evaluation, and the results are summarized in Tables 4 and 5. Prophylactic clipping was performed for 38 lesions (25%) but not for 112 lesions (75%). A 10‐mm snare was used for six lesions (4%) and a 15‐mm snare for 144 lesions (96%). Patients who received a prophylactic clip were more frequently taking antithrombotic agents than those who did not receive a prophylactic clip (*p* = 0.037). No bleeding, perforation, or other adverse events were observed in either the prophylactic clip or non‐prophylactic clip group. No significant differences in treatment outcomes or pathological findings were observed between the two groups. However, the submucosal layer tended to be thicker in the 15‐mm snare group.

**TABLE 4:** Baseline characteristics, treatment outcomes, and pathological evaluation according to prophylactic clip.

	Prophylactic clip	Non‐prophylactic clip	
Patients	*n* = 31	*n* = 64	*p*‐Value
Age, years (IQR)	77 (73.5–80)	72.5 (67–78)	0.019
Male sex, *n* (%)	24 (77)	50 (78)	1.00
Antithrombotic agents, *n* (%)	11 (35)	10 (16)	0.037

Lesions	*n* = 38	*n* = 112	
Size, mm (IQR)	7 (6–8)	7 (6–8)	0.371
Location, *n* (%)			0.221
Cecum	6 (16)	4 (3.6)	
Ascending colon	8 (21)	24 (21)	
Transverse colon	7 (18)	31 (28)	
Descending colon	4 (11)	13 (12)	
Sigmoid colon	12 (32)	34 (30)	
Rectum	1 (2.6)	6 (5.4)	
Macroscopic type, *n* (%)			1.00
0‐Is	33 (87)	97 (87)	
0‐IIa	5 (13)	15 (13)	
Treatment outcomes	*n* = 38	*n* = 112	
Procedure time, seconds (IQR)	62 (47–85)	66 (53–91)	0.511
Gastrointestinal fellows performed, *n* (%)	8 (21)	40 (36)	0.110
En bloc resection, *n* (%)	38 (100)	112 (100)	N/A
R0 resection, *n* (%)	29 (76)	95 (85)	0.320
Sufficient vertical R0 resection (SVR0), *n* (%)	26 (68)	89 (80)	0.186
Adverse events, *n* (%)	0 (0)	0 (0)	N/A

Abbreviations: IQR, interquartile range; LPPC‐HSP, hot snare polypectomy with low‐power pure‐cut current. N/A, not available.

**TABLE 5:** Treatment outcomes and pathological evaluation according to snare size.

Snare	10 mm	15 mm	*p‐*Value
Lesions	*n* = 6	*n* = 144	
Size, mm (IQR)	7.5 (7–8)	7 (6–8)	0.354
Location, *n* (%)			0.947
Cecum	0 (0)	10 (6.9)	
Ascending colon	1 (17)	31 (22)	
Transverse colon	2 (33)	36 (25)	
Descending colon	0 (0)	17 (12)	
Sigmoid colon	3 (50)	43 (30)	
Rectum	0 (0)	7 (4.9)	
Macroscopic type, *n* (%)			1.00
0‐Is	6 (100)	124 (86)	
0‐IIa	0 (0)	20 (14)	
Procedure time, seconds (IQR)	77 (61–102)	65 (50–91)	0.539
Gastrointestinal fellows performed, *n* (%)	0 (0)	48 (33)	0.177
Completion of LPPC‐HSP, *n* (%)	6 (100)	144 (100)	N/A
En bloc resection, *n* (%)	6 (100)	144 (100)	N/A
Adverse events, *n* (%)	0 (0)	0 (0)	N/A
Snare	10 mm	15 mm	*p*‐value
Pathological evaluation	*n* = 6	*n* = 144	
R0 resection, *n* (%)	4 (67)	120 (83)	0.278
Sufficient vertical R0 resection (SVR0), *n* (%)	4 (67)	111 (77)	0.624
Presence of submucosal tissue within the resected specimen, *n* (%)	4 (67)	128 (89)	0.153
	*n* = 6	*n* = 140	
Maximum thickness of submucosal tissue within the resected specimen, µm (IQR)	864 (204–938)	1398 (742–2054)	0.161
Thickness of submucosal tissue within the resected specimen at the center, µm (IQR)	647 (120–913)	1283 (638–2039)	0.164
Thickness of submucosal tissue within the resected specimen at the margin, µm (IQR)	712 (144–896)	851 (511–1286)	0.354

Abbreviations: LPPC‐HSP, hot snare polypectomy with low‐power pure‐cut current; IQR, interquartile range; N/A, not available;

## 4 Discussion

This study is the first to evaluate the vertical resection ability of LPPC‐HSP for nonpedunculated colorectal polyps smaller than 10 mm, including the containing submucosal tissue rate and thickness of the submucosal tissue. In this study, an acceptable SVR0 rate was achieved, demonstrating that sufficient submucosal tissue could be reliably resected even with a low‐power pure‐cut current. These findings suggest that LPPC‐HSP may be applicable to colorectal polyps smaller than 10 mm that are potentially malignant, similar to conventional resection methods with electrocautery.

The study demonstrated a favorable SVR0 rate, and the lower limit of the 90% CI exceeded the predefined threshold of 60%. Thus, the primary endpoint was achieved. This suggests that the resection ability of LPPC‐HSP for small polyps is not inferior to that of conventional resection methods with electrocautery. When high‐grade adenomas or carcinomas are suspected, it is recommended to use electrocautery during endoscopic resection procedures. A previous study [20] reported an SVR0 rate of 65.6% with underwater EMR (UEMR) for duodenal neoplasms and concluded that UEMR should be performed when low‐grade adenomas cannot be diagnosed with high confidence. In our study, the SVR0 rate of LPPC‐HSP was comparable to this finding, suggesting that LPPC‐HSP may be applicable for small polyps with potential malignancy.

In the pathological evaluation, specimens resected by LPPC‐HSP included the muscularis mucosa in all cases and submucosal tissue in 132 lesions (88%), with a median maximum submucosal thickness of 1331 µm, indicating that LPPC‐HSP enables accurate pathological evaluation due to adequate submucosal tissue acquisition and thus has acceptable vertical resection ability. Treatment outcomes reported in previous studies [13, 15, 24], including those of conventional HSP and EMR for colorectal polyps smaller than 10 mm and LPPC‐HSP for 10–14 mm colorectal polyps, are summarized in Table 6, along with the results of our study. According to previous reports [13], the rate of containing submucosal tissue achieved by CSP is limited to 24%. In contrast, the rate of containing submucosal tissue with LPPC‐HSP in our study was 88%, demonstrating a superior vertical resection depth compared with CSP. This rate of LPPC‐HSP in our study was comparable to that of conventional HSP (81.5%) [13] and EMR (92%) [24] for small colorectal polyps, as well as that of LPPC‐HSP for 10–14 mm polyps (88.8%) [15], suggesting that LPPC‐HSP for nonpedunculated colorectal polyps smaller than 10 mm can similarly achieve adequate vertical resection comparable to these conventional techniques. Compared with the submucosal thickness reported in previous studies on CSP, HSP, and EMR, the submucosal thickness in our study tended to be thicker. These results may be attributable to several factors. First, the method used to measure submucosal thickness differed from that used in some previous studies. In our study, submucosal thickness was calculated simply as the distance between the resection margin and the muscularis mucosa, similar to the method used by Suzuki et al. [13], whereas Ito et al. [24] measured it based on the area and distance from the muscularis mucosa. Moreover, the submucosal thickness may be influenced by the specimen fixation and sectioning technique during pathological preparation and should be interpreted with caution. Although the difference was not statistically significant in Table 5, specimens resected with the 15‐mm snare tended to include a thicker submucosal tissue than those resected with the 10‐mm snare. In addition, we also performed air deflation before snaring so that the lesion could be captured together with the surrounding normal mucosa. Both factors may influence the depth of submucosal thickness. However, a previous animal study suggested that the resection depth achieved with LPPC‐HSP is limited to the superficial submucosa [15]. Therefore, this technique should be avoided for lesions with endoscopic findings suggestive of submucosal invasive cancer.

**TABLE 6:** Comparison of the thickness of submucosal tissue between hot snare polypectomy with low‐power pure‐cut current (LPPC‐HSP), cold snare polypectomy (CSP), conventional hot snare polypectomy (HSP), and endoscopic mucosal resection (EMR).

	Our study	Ito et al. [24]		Suzuki et al. [13]		Imai et al. [15]
Lesion size	1–9 mm	1–9 mm		1–10 mm		10–14 mm
Method	LPPC‐HSP	CSP	EMR	CSP	HSP	LPPC‐HSP
R0 resection rate	83.0%	43.0%	62.0%	77.3%	92.3%	85.7%
The containing muscularis mucosa rate	100.0%	91.0%	N/A	92.0%	96.3%	100%
The containing submucosal tissue rate	88.0%	8.9%	92.1%	24.0%	81.5%	88.8%
Thickness of the submucosal tissue within the resected specimen µm, median (IQR)	1331 (736–2044)	76 (44–127)	338 (237–468)	0 (N/A)	600 (N/A)	N/A
Measurement of submucosal thickness	measured perpendicular to the muscularis mucosa	(area)/(length of muscularis mucosa)		measured perpendicular to the muscularis mucosa		N/A
Snare	10 or 15 mm	10 mm	N/A	10 mm	10 mm	10 or 15 mm

Abbreviations: CSP, cold snare polypectomy; EMR, endoscopic mucosal resection; HSP, hot snare polypectomy; IQR, interquartile range; LPPC‐HSP, hot snare polypectomy with low‐power pure‐cut current; N/A, not available; area; area of muscularis mucosa + submucosa.

In terms of adverse events, no bleeding, perforation, or other serious adverse events were observed, indicating that LPPC‐HSP may be a safe procedure. Previous studies reported delayed bleeding rates of 0.4% with CSP and 1.5% with conventional HSP [6], whereas LPPC‐HSP was associated with delayed bleeding rates of 0.6% per patient (2/339) and 0.5% per lesion (2/410) [21]. These findings suggest that despite endoscopic resection with electrocautery, the safety of LPPC‐HSP may be similar to that of CSP. LPPC‐HSP uses a low‐power pure cutting current, which may reduce thermal injury to the muscular layer compared with conventional coagulation or blended currents [15, 25]. Although LPPC‐HSP achieves a deeper depth of submucosal thickness than CSP, its delayed bleeding rate remains similarly low. These findings indicate that injury to the residual submucosa, rather than resection depth, may be a more important determinant of delayed bleeding. Furthermore, LPPC‐HSP may reduce injury to deeper submucosal vessels and the muscular layer, potentially reducing the risk of delayed bleeding and perforation. Patients who received a prophylactic clip were more frequently taking antithrombotic agents than those who did not receive one (Table 4), suggesting that the prophylactic clip may have influenced the occurrence of adverse events. Although a prophylactic clip takes extra time, LPPC‐HSP is relatively simple, since submucosal injection is unnecessary. A prophylactic clip does not reduce delayed bleeding in small polyps [26] and is generally effective for large polyps [27]; therefore, it may not be necessary for every lesion. However, the current evidence remains limited by the small sample size, and further multicenter prospective studies are required to evaluate the safety of LPPC‐HSP and to define appropriate indications for prophylactic clipping.

This study has several limitations. First, this was a single‐center, single‐arm, observational study. Because no direct comparison with conventional techniques, such as CSP, conventional HSP, and EMR, was performed, multi‐center randomized controlled studies are required to determine the generalizability of these findings. Second, the proportion of lesions with advanced histology was relatively low. We performed LPPC‐HSP whenever endoscopic findings suggestive of advanced histology [10], as described in the Methods, were observed. Although these endoscopic findings have a high negative predictive value and are therefore useful for excluding advanced histology, their sensitivity has been reported to be only 20%–30% [10], even in the high‐volume center in which they were originally investigated. Because we applied LPPC‐HSP to any lesion with even a slight suspicion of advanced histology, this is likely the main reason for the low proportion of lesions with advanced histology in our cohort. However, LPPC‐HSP is minimally invasive, and the delayed bleeding rate was 0.5% among 410 lesions [21]. Therefore, the disadvantage of performing LPPC‐HSP for lesions that could otherwise be treated by CSP might be small. Further studies are warranted to confirm this. Third, long‐term outcomes such as recurrence were not evaluated in the present study. Accordingly, the evaluation of the long‐term outcomes of LPPC‐HSP in future studies is necessary.

In conclusion, the vertical resection ability of LPPC‐HSP was acceptable. LPPC‐HSP may be applicable to colorectal polyps smaller than 10 mm that are potentially malignant, similar to conventional resection methods with electrocautery.

## Author Contributions

**Conception and design**: Hidenori Kimura and Ryo Mizuno. **Drafting of the manuscript**: Hidenori Kimura and Ryo Mizuno. **Revision of the article**: Ryoji Kushima, Akifumi Notsu, Atsushi Nishida, and Osamu Inatomi. **Final approval of the article**: Takuji Iwashita.

## Funding

All authors disclosed no financial relationships. This study was supported by the JSPS KAKENHI (Grant 25K21606).

## Ethics Statement

**Approval of the research protocol by an Institutional Reviewer Board**: This study was approved by the Institutional Review Board of Shiga University of Medical Science (Institutional number R2024‐013).

## Consent

Written informed consent was obtained from all participants.

## Conflicts of Interest

Takuji Iwashita is an editorial board member of DEN Open. The other authors declare no conflicts of interest.

## Data Availability

The data that support the findings of this study are available from the corresponding author upon reasonable request.
